# Biological age and environmental risk factors for dementia and stroke: Molecular mechanisms

**DOI:** 10.3389/fnagi.2022.1042488

**Published:** 2022-12-22

**Authors:** Pablo Knobel, Rachel Litke, Charles V. Mobbs

**Affiliations:** ^1^Department of Environmental Medicine and Public Health, Icahn School of Medicine at Mount Sinai, New York, NY, United States; ^2^Nash Family Department of Neuroscience, Friedman Brain Institute, Icahn School of Medicine at Mount Sinai, New York, NY, United States

**Keywords:** dementia, stroke, aging, environmental risk factors, epigenetic effects

## Abstract

Since the development of antibiotics and vaccination, as well as major improvements in public hygiene, the main risk factors for morbidity and mortality are age and chronic exposure to environmental factors, both of which can interact with genetic predispositions. As the average age of the population increases, the prevalence and costs of chronic diseases, especially neurological conditions, are rapidly increasing. The deleterious effects of age and environmental risk factors, develop chronically over relatively long periods of time, in contrast to the relatively rapid deleterious effects of infectious diseases or accidents. Of particular interest is the hypothesis that the deleterious effects of environmental factors may be mediated by acceleration of biological age. This hypothesis is supported by evidence that dietary restriction, which universally delays age-related diseases, also ameliorates deleterious effects of environmental factors. Conversely, both age and environmental risk factors are associated with the accumulation of somatic mutations in mitotic cells and epigenetic modifications that are a measure of “biological age”, a better predictor of age-related morbidity and mortality than chronological age. Here we review evidence that environmental risk factors such as smoking and air pollution may also drive neurological conditions, including Alzheimer’s Disease, by the acceleration of biological age, mediated by cumulative and persistent epigenetic effects as well as somatic mutations. Elucidation of such mechanisms could plausibly allow the development of interventions which delay deleterious effects of both aging and environmental risk factors.

## Introduction

### The transition from infectious diseases to chronic age-related diseases

The dramatic increase in life expectancy, even when excluding infant mortality, from about 40 years in 1900 to about 80 years today, is mainly due to the over 90% decline in deaths due to infectious diseases (Armstrong et al., 1999). Today, most morbidity and mortality are due to chronic diseases, especially neurological conditions (Armstrong et al., 1999). The prevalence of chronic diseases increases with age, while infectious disease incidence does not (Armstrong et al., 1999). In a representative study in Rhode Island, dementia and stroke were the second and fourth leading cause of crude mortality, the third and fourth leading causes of disability-adjusted life years, the fourth and fifth leading causes of years of life lost, and the third and fourth leading causes of years lived with disability (Katan and Luft, 2018). Alzheimer’s Disease (AD) alone has been estimated to cost the US health system $305 billion in 2020, and costs are expected to reach over $1 trillion per year by 2050 if effective treatments are not developed https://doi.org/10.37765/ajmc.2020.88482. By way of comparison, the entire budget for Medicare (which would be expected to cover the vast majority of health care for dementia) was “only” $767 billion (13% of the entire federal budget) in 2020.^1^ Similarly, stroke is the second leading cause of death and a major cause of disability worldwide (Katan and Luft, 2018). Thus, discovering interventions to reduce the incidence and improve the treatment of neurological conditions could reduce the burden of these diseases, potentially saving Medicare from bankruptcy, among many other benefits.

### Environmental risk factors for dementia and other cognitive impairments

Disparities in the risk of dementia are largely due to environmental factors that are differentially experienced by different ethnic groups (Deckers et al., 2019). In this review we use the term “environmental risk factors” to refer any feature of the environment except for agents of infectious diseases. However, as we discuss below in addition to causing well-known short-term deleterious effects, infectious diseases can sometimes also cause long-term chronic conditions similar to those characteristic of aging, and this phenomenon can inform mechanisms by which standard risk factors produce similar symptoms. Several studies have found associations between the built and social environment and age-related outcomes (Clarke and Nieuwenhuijsen, 2009). Greenness and walkability were associated with better mental health, cognitive function, wellbeing, and physical capability among older adults (Markevych et al., 2017; Nieuwenhuijsen et al., 2017; de Keijzer et al., 2020). Safety has been particularly noted as crucial for the health of the elderly population (Tong et al., 2016), with associations between self-reported safety and self-reported health (Cain et al., 2018). Lifestyle patterns – including diet and physical activity – were influenced by the built and social environment and influence age-related health (Pruchno and Wilson-Genderson, 2012; Pruchno R. A. et al., 2012; Pruchno R. et al., 2012).

Brain structure is mainly formed during early life (Lenroot and Giedd, 2006) and is a crucial determinant of individuals’ susceptibility to AD (Borenstein et al., 2006). The environmental factors described above have been associated with children’s neurodevelopment. Socioeconomic status has been linked to diverse neurocognitive outcomes (Ursache and Noble, 2016). Greenness benefits neuropsychological outcomes and mental health (Luque-Garcia et al., 2022). High-risk neighborhoods – defined with a composite index including neighborhood measures such as percentage of the population with high school diploma, percentage of a single-parent household, and racial composition – were associated with increased odds of severe neurodevelopmental impairment, cognitive and language delays (Nwanne et al., 2022).

Healthy prenatal and childhood diet has been linked to improved neurodevelopment and improved childhood health outcomes such as body composition (Nigg et al., 2016). In addition, the food environment has been associated with executive functioning in children (Bryant et al., 2020). Lower objective food store availability has also been associated with increased dementia incidence, possibly mediated by reduced physical activity as much as diet (Tani et al., 2019). In another study, participants who reported low subjective food store availability had a higher likelihood of developing disability than those who reported high subjective food store availability, and low subjective food store availability was associated with early onset of disability (Wu et al., 2020). On the other hand, some fast-food establishments have been associated with improved cognitive functioning: the authors hypothesize that this surprising result could be due to the benefits of social interactions (Finlay et al., 2020).

Elucidation of the interaction of environmental factors and age on neurological diseases also promises to address another major challenge to the health system: the unconscionable persistence of major health disparities in these diseases as a function of ethnicity. For example, it is estimated that individuals of African-American heritage are about twice as likely to develop AD, and those of Hispanic heritage about 50% more likely, than individuals of Caucasian Western European heritage. These disparities are not due to genetic differences but rather to different environmental factors that different ethnic groups experience because of segregation due to a history of structural racism (Rajan et al., 2021). The prevalence of stroke exhibits similar disparities between ethnic groups which are similarly due to environmental factors (Levine et al., 2020). Thus identifying and eliminating these environmental discrepancies could rectify what is clearly among the most egregious failures of the American health system.

## Do environmental risk factors accelerate biological age?

The CDC defines the term “chronic disease” as a condition that lasts one year or more and require ongoing medical attention or limit activities of daily living, or both. Conceptually it may be useful to use the term “acute disease” to refer to conditions in which symptoms last less than a year (and usually less than a month). This distinction, for example, provides a framework for examining the mechanisms by which COVID-19 evolves from an acute disease to a chronic disease with entirely different symptoms (Higgins et al., 2021). Indeed, the most fastidious clinicians diagnose post-acute (or long) COVID syndrome only if acute symptoms (such as acute respiratory distress) are not evident (Lechner-Scott et al., 2021; Tabacof et al., 2022). Other viral diseases also present acute and chronic (post-acute) forms, such as (acute) Lyme Disease followed by (post-treatment) Lyme disease syndrome (PTLDS) (Wong et al., 2022). SARS-CoV1 and MERS exhibit similar acute and post-acute forms (Higgins et al., 2021). It is of interest that cognitive impairments are a particularly prominent feature of post-acute COVID syndrome (Lechner-Scott et al., 2021; Tabacof et al., 2022).

Furthermore, evidence suggests that COVID-19 activates mechanisms that are implicated in the etiology of AD. For example, chronic conditions such as diabetes are risk factors for both COVID-19 and AD and may exacerbate the symptoms of both conditions (Xia et al., 2021). The relevant mechanism mediating these effects is most likely increased inflammation, particularly TNF-alpha and IL-6, which appear to be among the most robust predictors of severity of disease and death due to COVID-19 (Del Valle et al., 2020). Similarly, TNF-alpha and IL-6 are highly implicated in the etiology of both AD (Cavanagh and Wong, 2018; De Sousa Rodrigues et al., 2019; Kaur et al., 2019; Park et al., 2019) and stroke (Mayne et al., 2001; King et al., 2013; Lei et al., 2013; Doll et al., 2015).

**FIGURE 1:**
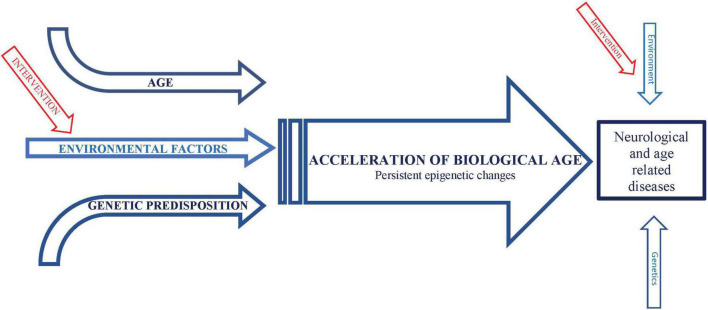
Schematic representation of conceptual framework integrating effects of environmental factors and age on neurological and age-related diseases.

A potentially informative interaction between infectious diseases and aging is suggested by the observation that mortality rate in the acute phase of COVID-19 increases exponentially with age (Levin et al., 2020). The most likely explanation for this striking phenomenon is that, as with other infection-induced mortality such as sepsis (Lv et al., 2014), the age-related increase in mortality from COVID-19 is most likely due to excessive inflammation (“cytokine storms”) in response to the infection. It is not understood why inflammation increases with age, a phenomenon referred to as “inflammaging” (Franceschi et al., 2018). However, extensive evidence suggests that “environmental and lifestyle factors can promote systemic chronic inflammation (SCI) that can in turn, lead to several diseases … such as neurodegenerative disorders” (Furman et al., 2019). Nevertheless, environmental factors are only a subset of several processes driving inflammaging. Similarly, inflammation is only a subset of mechanisms mediating effects of environmental factors to drive age-related neurological conditions, some of which may be direct (Figure 1). Consistent with the likely role of TNF-alpha and IL-6, these cytokines increase during normal aging, linked with age-associated increased risk of morbidity, including neurological conditions, and mortality (Michaud et al., 2013). Particularly compelling evidence of a link between environmental risk factors, inflammation, and aging is that protective effects of dietary restriction, which generally ameliorates age-related impairments, including in models of AD (Halagappa et al., 2007; Wu et al., 2008; Zhang et al., 2009) and stroke (Manzanero et al., 2011), appear to be mediated by a reduction in innate immune system activity, the source of inflammation, via a metabolic mechanism (Wu et al., 2019). This metabolic mechanism is almost certainly a shift away from glycolysis and toward the use of alternative substrates, which is a universal effect of dietary restriction and is thought to mediate the protective effects of dietary restriction (Mobbs et al., 2007; Mobbs, 2018). This hypothesis is particularly compelling for AD and stroke, since the activation of microglial inflammation in these conditions by Abeta is associated with the reverse metabolic profile produced by dietary restriction: increased glycolysis and reduced oxidative phosphorylation (Baik et al., 2019). In fact, increased glycolysis is required for microglia to produce inflammatory responses which drive neuronal death (Meng et al., 2020). Major support that inflammation causes, and is not simply caused by, age-related diseases, is that Humira, simply a monoclonal antibody against TNF-alpha, is the best-selling drug in history and is increasingly the first-in-line therapy for a wide range of age-related conditions (Ladika, 2019).

The observation that mortality due to COVID-19 increases exponentially with age, plausibly due at least in part to an age-related increase in inflammatory responses, raises the critical question of why inflammation increases with age. This tendency is part of a wider phenomenon described as “biological age” as opposed to chronological age (Polidori et al., 2021). To the extent that increased inflammation may be considered a component of biological age, this raises the hypothesis that environmental factors may accelerate biological age. Thus elucidation of these mechanisms raises the tantalizing possibility that single interventions focused on delaying the progression of biological age could also generally delay deleterious effects of environmental factors, similar to the generally protective effects of dietary restriction, which arguably does precisely this: delay the progression of biological age. While this may seem like a wildly speculative hypothesis, herein we will develop arguments in favor of this and even propose specific mechanisms driving biological age in the context of deleterious environmental factors.

In fact, lingering chronic symptoms after the acute phase of infectious diseases, which have more in common with chronic diseases than acute diseases, do appear to entail accelerated biological aging (Mongelli et al., 2021). To address this hypothesis, it is necessary to elucidate mechanisms by which effects of these chronic processes accumulate and persist. The issue addressed herein is whether similar mechanisms mediate the deleterious effects of both environmental factors and age.

Arguably the first convincing demonstration of the cumulative effects of a deleterious environment on chronic disease was the observation that smoking is by far the most important risk factor for lung cancer (Doll, 1953). Subsequently, mechanisms mediating the cumulative deleterious effects of smoking were elucidated: smoking, like all carcinogens, causes the accumulation of somatic mutations in lung cells, and when sufficient mutations accumulate in oncogene and anti-oncogene genes, the cells transform to a cancerous phenotype (Yoshida et al., 2020). This basic mechanism was further supported by the discovery that carcinogens are generally powerful mutagens as well (Wagner et al., 1992). Similar observations have been made across a wide variety of tissues and associated with other environmental factors. For example, RNA-sequencing data from ∼6,700 samples across 29 normal tissues demonstrated many somatic mutations which increase with age, and sun-exposed skin, esophagus, and lung had a higher mutation burden than other tested tissues, further supporting that environmental factors can promote somatic mutations which increase with age (Yizhak et al., 2019). Specifically relevant to age-related neurological and psychiatric diseases, known to be influenced by environmental factors such as urban living, somatic mutations also increase with age in the brain, suggesting that environmental factors drive those age-related mutations as well (Bedrosian et al., 2016). More recent studies have demonstrated that persistent effects of carcinogens leading to carcinogenesis may also involve persistent modulation of epigenetic states (e.g., DNA methylation and histone modification) that do not entail mutations in the primary DNA sequence (Peltomaki, 2012). Air pollution, the main environmental risk factor for dementia (Killin et al., 2016), also produces persistent changes DNA methylation (Bollati and Baccarelli, 2010). As described in detail below, epigenetic changes in DNA methylation are now the key parameters used to calculated biological age, and these epigenetic changes are highly sensitive to environmental factors (Oblak et al., 2021).

Since deleterious effects of environmental factors on chronic diseases are only manifest after enough time has passed for effects to accumulate (e.g., development of sufficient numbers of mutations and persistent epigenetic modifications), it could be argued that the correlation between age and age-related diseases is simply due to the accumulation of environmental insults, even if the nature of those environmental factors may not generally be known. In fact, this is essentially the argument developed by the same investigator, Richard Doll, who discovered the relationship between smoking and lung cancer. This perspective was developed in a review provocatively entitled “There is no such thing as aging”, written some 40 years after his discovery of the link between smoking and lung cancer (Peto and Doll, 1997). This review develops the hypothesis that the link between age and cancer is straightforward: even without carcinogen activity, since DNA replication is imperfect, cells will accumulate mutations during mitosis as a matter of random factors, essentially as a consequence of the Second Law of Thermodynamics. While this is a plausible view of the relationship between age and cancer, other age-related diseases such as AD are largely manifest in non-dividing cells (e.g., neurons) so there is no reason that these diseases would be driven by the same mechanisms driving cancer, other than that damage driving such diseases are similarly a result of the time it takes for sufficient entropy to increase to produce the disease (again, Second Law of Thermodynamics). Thus the review concludes that “Similar considerations probably also apply to a wide range of adult diseases: the fact that they tend to arise in the same part of the life span is not good evidence that they have similar underlying mechanisms, nor is it good evidence that any single, unifying change awaits discovery that could properly be called ‘aging”’ (Peto and Doll, 1997).

On the other hand, the review did concede that “although there may be no direct link between any one thing that can usefully be called ‘aging’ and the rates of the separate cellular processes that culminate in cancer, there remains a strong and mechanistically unexplained relation between the life span and the rates of these processes” and without that link “humans would not survive” (Peto and Doll, 1997). Similarly unexplained is the remarkably robust exponential increase in mortality with age, the so-called Gompertz equation, such that the log transform of age-dependent mortality yields a straight line whose slope (designated “G”) varies widely between species and indeed could be considered a mathematical definition of the rate of aging (Olshansky and Carnes, 1997). Of particular interest in this regard are manipulations that robustly increase lifespan and delay age-related diseases. These include dietary restriction (Neafsey et al., 1989; Yen and Mobbs, 2008), reduction of insulin-like signaling (Johnson, 1990), and reduced temperature (Yen and Mobbs, 2009), all reduce the slope of G, strongly implying common mechanisms that determine lifespan impacted by these manipulations and the development of age-related diseases (which can be defensibly referred to as “aging”). Indeed, it has been proposed that only diseases whose incidence, like mortality, increases exponentially with age, which includes “the major non-communicable diseases dementia, stroke and ischemic heart disease,” should be considered age-related diseases. However, this defensible position implicitly assumes that the same processes which drive the exponential increase in mortality also drives the exponential increase in these diseases (Le Couteur and Thillainadesan, 2022).

## Does biological age influence the cumulative effects of environmental factors on life expectancy and age-related diseases?

Further support that lifespan and diseases of aging are driven by common mechanisms is the discovery of robust markers of “biological age,” particularly epigenetic modifications, which correlate with chronological age, but better predict life expectancy and age-related diseases. There are four main related measures of biological age, the Hannum, Horvath, Levine, and Grimage “clocks,” all of which, while correlated with chronological age, predict life expectancy and morbidity significantly better than chronological age (Oblak et al., 2021). It is therefore of interest that COVID-19 accelerates biological age, as indicated both by standard epigenetic modifications of DNA methylation as well as telomerase shortening in blood cells (Mongelli et al., 2021), and evidence that the exponential increase of mortality with age from COVID-19 correlates better with biological age than chronological age (Polidori et al., 2021). Of particular interest to the present theme, these 4 “clocks” are accelerated by a variety of environmental risk factors; smoking has by far the greatest effect on accelerating these “clocks”, followed by alcohol (Oblak et al., 2021). These results quantitatively track with the relative risk in global cancer produced by smoking and alcohol (Collaborators, 2022).

An improved model showed that biological age at baseline was superior to chronological age and traditional biomarkers, in predicting mortality, morbidity and onset of specific diseases such as stroke, cancer, and diabetes (Waziry et al., 2019). Furthermore, adding brain biomarkers for neurological degeneration (plasma NfL, total-tau, amyloid beta–40 and –42) further improved the association of biological age with dementia, including AD These observations were confirmed, and extended by even further refinement of the markers of biological age (Wu et al., 2021).

To the extent that environmental risk factors accelerate biological age, a salient issue is whether this acceleration is influenced by biological age itself. For example, since low birth weight is a risk factor for many conditions that are mainly manifest during aging, it is plausible that deleterious effects of environmental factors during fetal development can persist long after the factors themselves are no longer acting on the organism and exacerbate impairments that occur during normal aging (Godfrey and Barker, 2001). This latter study raises the critical question of whether cumulative effects of environmental factors accumulate at a constant rate, or whether effects may depend on age or prior exposure to the environmental factors and may be influenced by environmental factors. For example, the environmental factor most implicated in the development of dementia is air pollution (Killin et al., 2016). It is therefore of great interest that fetal exposure to air pollution produces epigenetic effects observable in the placenta, which if also produced in the fetus during development would be expected to persist into adulthood, thus predisposing to impaired health in adulthood and aging (Saenen et al., 2019).

## Molecular hysteresis occurs in response to environmental factors and impacts biological age

The particular sensitivity of the fetus to environmental factors is reminiscent of the more general phenomenon of “critical periods,” in which the fetus, neonate, and even juvenile is particularly susceptible to environmental perturbation, which has consequences in the adult. For example, the default sex during mammalian fetal development is female; the fetus with a Y chromosome will only develop into a male if it is exposed to androgens during fetal development, although paradoxically, this effect occurs via the conversion of androgens to estrogens (Gorski, 2002). However, mammals are not completely mature when born, so some amount of sexual maturation continues between birth and weaning. During this critical period, treating neonates with relatively low doses of estradiol will not masculinize them, but it will render them permanently acyclic and sterile (Mobbs et al., 1984). Such low doses of estradiol no longer produce sterility after weaning, but a single injection of a very high dose of estradiol will produce permanent sterility due to hypothalamic defects which are similar to those hypothalamic defects that seem to be the cause midlife reproductive failure in mice (Mobbs, 1994). Conversely, removing ovaries from young mice prevents the age-related hypothalamic defects (Mobbs, 1994). These observations and related studies led to the hypothesis that each time estradiol-regulated genes were acted on during normal reproductive cycling, there remain residual effects of that regulation, which thereby accumulate to produce largely irreversible effects on gene expression (Mobbs, 1994). A similar phenomenon occurs with estrogen-induced ovalbumin, in which a single exposure to estradiol produces permanent epigenetic changes, indicated by persistently increased DNAse sensitivity of the gene associated with persistently enhanced sensitivity to estradiol (Burch and Weintraub, 1983). A similar “gene memory” effects occur in the lac operon (Laurent et al., 2005; Mobbs et al., 2007). Glucose induces a similar persistent effect on glucose-regulated gene expression also associated with persistent epigenetic changes in glucose-regulated genes, a phenomenon referred to as “metabolic memory” and which is thought to mediate the progression of diabetic complications, and in particular why diabetic complications appear irreversible even when blood glucose is completely normalized by beta cell transplants (Ihnat et al., 2007; Kowluru et al., 2007, 2010; Villeneuve et al., 2008; Siebel et al., 2010; Tonna et al., 2010; Zhong and Kowluru, 2010; Kim et al., 2013; Mobbs, 2018). It is thus particularly informative that diabetes, which as described above is a major risk factor for both AD and stroke, accelerates biological age as determined by the classic “clock” models (Oblak et al., 2021). Similarly informative, these persistent effects of glucose are associated with epigenetic modifications which increase expression of, or sensitize expression of, inflammatory genes (Zheng et al., 2021). Cumulative glucose-induced persistent epigenetic modifications also appear to drive many aspects of the aging process, and the many protective effects of dietary restriction are arguably mediated by a chronic reduction of glucose, and thus a delay in the cumulative epigenetic effects of glucose which may drive many age-related diseases, including neurological diseases (Mobbs, 2018).

We hypothesize that a similar phenomenon of “gene memory” or “molecular hysteresis” occurs in response to environmental risk factors, consistent with evidence that chronic exposure to environmental factors increase expression of, or potentiate priming of, inflammatory genes, or responses to inflammatory cytokines, particularly TNF-alpha (Kalliolias and Ivashkiv, 2016). Furthermore, stimulation of inflammatory activity, particularly TNF-alpha secretion, is associated with induction of HDAC activity, inhibition of which reduces these epigenetic modulations and secretion of TNF-alpha (Doherty et al., 2013). This is particularly relevant because we have previously demonstrated that protective effects of dietary restriction as well as inhibition of the daf-2/Insulin IGF1/FOXO pathway on lifespan and impairments in an animal model of AD require the histone acetyltransferase CBP, and these protective effects are mimicked by HDAC inhibition (Zhang et al., 2009). Taken together, these data support that exposure to environmental risk factors as well as during aging (possibly due to the cumulative effects of glucose) promote persistent increase in inflammatory gene expression via epigenetic modifications characteristic of biological age, which in turn drives many if not all, impairments associated with environmental risk factors and age, including dementia and stroke (Figure 1).

## Author contributions

PK, RL, and CM: literature review, writing – original draft preparation, and reviewing and editing. CM: funding acquisition and supervision. All authors contributed to the article and approved the submitted version.

## Footnotes

1
https://www.pgpf.org/budget-basics/medicare
